# Supplementary material for: The scale and dynamics of COVID-19 epidemics across Europe
Source: R Soc Open Sci. 2020 Nov 25;7(11):201726. doi: 10.1098/rsos.201726 (PMC7735356; doi:10.1098/rsos.201726)
Supplement: Supplementary Materials [file rsos201726supp1.docx]

Supplementary Materials for

**The scale and dynamics of COVID-19 epidemics across Europe**

Christopher Dye, Russell C.H. Cheng, John S. Dagpunar & Brian G. Williams

Correspondence to: [christopher.dye@zoo.ox.ac.uk](mailto:christopher.dye@zoo.ox.ac.uk)

**This PDF file includes:**

Materials and Methods

Supplementary Text

Figs. S1 to S6

Tables S1 to S3

Materials and Methods

This supplement to the main text provides data sources, methods of data analysis, the mathematical model of COVID-19 dynamics, plus additional data and Figures. All data and models used in this study can be found in the Dryad Digital Repository at <https://datadryad.org/stash/dataset/doi:10.5061/dryad.f1vhhmgv6> [1].

**Data sources**

This analysis is based on COVID-19 deaths reported from 30 European countries [2] for which the ISO 3166-1 alpha-3 abbreviations are in Table S1. Like other investigators [3], we used reported deaths because they are likely to be more accurate than reported cases, both in absolute magnitude (scale of the epidemic) and in the distribution of deaths through time (shape or dynamics of the epidemic). The information on daily deaths is compiled by the European Centre for Disease Prevention and Control using standard methods applied to all countries [2].

Although the completeness of death reports has been questioned, the clear patterns of association in Figs 3 and 4 of the main text suggest that both the data, and the empirical model (skew-logistic) used to describe the data, portray real effects, rather than artefacts, of COVID-19 epidemics across the 30 countries included in this investigation.

**Table S1**. Three-letter ISO codes for the 30 countries included in this analysis (Figs 4a, S5, S6).

| Albania | ALB | Italy | ITA |
| --- | --- | --- | --- |
| Austria | AUT | Luxembourg | LUX |
| Belgium | BEL | Netherlands | NLD |
| Bulgaria | BGR | Norway | NOR |
| Croatia | HRV | Poland | POL |
| Czech Republic | CZE | Portugal | PRT |
| Denmark | DNK | Romania | ROU |
| Finland | FIN | Serbia | SRB |
| France | FRA | Slovak Republic | SVK |
| Germany | DEU | Slovenia | SVN |
| Greece | GRC | Spain | ESP |
| Hungary | HUN | Sweden | SWE |
| Iceland | ISL | Switzerland | CHE |
| Ireland | IRL | Turkey | TUR |
| Israel | ISR | United Kingdom | GBR |

**SEIR model of COVID-19 transmission dynamics**

Fig 1 of the main text was constructed with a compartmental model of SARS CoV-2 transmission, framed in ordinary differential equations, and representing a homogeneously mixing population divided among those who are susceptible, exposed, infectious and recovered or died (SEIR), as follows:

I

S

E

R

$$\beta\mathrm{SI}$$

$\lambda E$

$$\mu I$$

**Fig S1**. The SEIR model. The compartments denote those in the population that are Susceptible, Exposed, Infected and Recovered.

The variables S, E, I and R satisfy the ordinary differential equations:

$\frac{dS(t)}{dt}=-\beta S\left( t \right)I(t)$ (1)

$\frac{dE}{dt}=\beta S\left( t \right)I\left( t \right)-\lambda E(t)$ (2)

$\frac{dI}{dt}=\lambda E\left( t \right)-\mu I(t)$ (3)

$\frac{dR}{dt}=\mu I(t)$ (4)

A convenient recent reference is Ma [4], though we have adjusted the notation here, reserving certain symbols for use in other standard ways.

$$p_{R}I$$

R\Z

Z

$(1-p_{R})$*I*

I

Z

**Fig S2**. Adjustment of the SEIR model where R is divided into two compartments, R \ Z, those that recover and Z, those that die; where $p_{R}$ is the proportion that recover.

As this discussion focuses on the number of people that die from COVID-19, the SEIR model is adjusted by splitting R, those that recover, to distinguish between Z, those that die, from those that survive. This arrangement is depicted in Fig S2, and is a simplified version of that used by Dagpunar [5] to consider the outcomes of hospitalization.

We allow this adjusted SEIR model to depend on certain parameters with the understanding that once these parameters are known the behaviour of the SEIR model is completely specified. The parameters of the model are described in Table S2, with parameter values estimated for Germany (Fig 1 of main text).

**Table S2**. Parameters of the SEIR model with estimates for Germany.

| Symbol | Definition | Value for Germany  in Fig 1 (95%CI) |
| --- | --- | --- |
| D = $\mu^{-1}$ | mean duration of infectiousness | 9.0 (7.9 – 9.9) days  or 5.0 or 4.0 (fixed) for comparison (Fig 1a) |
| T $=\beta^{-1}$ | mean interval between infectious contacts | 3.0 (2.8 – 3.3)  *R_0_* = $\beta$/μ = D/T |
| M$=\lambda^{-1}$ | mean latent period | 4.0 (2.5 – 5.1) days |
| $t_{0}$ | number of days from start of epidemic before observations began | 33 days (fixed) |
| $e_{0}$ | initial number of exposed | 9.2 (7.0 – 10.5) E-05 |
| $s_{0}$ | initial size of exposed population (to scale the epidemic) | 1.09 (1.07 – 1.15) E+06  or 1.5 or 0.5 E+06 for comparison (Fig 1b) |
| $\sigma$ | standard deviation of observational error | 41.9 (34.1 – 44.9) |
| $p_{R}$ | probability of recovering | 0.991 (fixed) |
| $\omega$ | mean time between the end of infectiousness and death | 11 days (fixed) |

For those who die from COVID-19, parameter $\omega$ specifies the time from the end of infectiousness to death (Fig S2, equation (4) so that:

$\frac{dZ}{dt}=\mu(1-p_{R})I(t-\omega)$ (5)

We denote by $\boldsymbol{\theta}\boldsymbol{=}\left( b1,b2,\ldots,b9 \right),$ the vector of parameters. With $\boldsymbol{\theta}$ given, the four differential equations (1), (2), (3) and (5) can be solved by numerical integration to give the trajectories

$S\left( t,\boldsymbol{\theta} \right), E\left( t,\boldsymbol{\theta} \right), I\left( t,\boldsymbol{\theta} \right),R\left( t,\boldsymbol{\theta} \right),Z\left( t,\boldsymbol{\theta} \right)$ for $t=1,2,\ldots,T$ (6)

where *t* is the day and *T* is the number of days of interest. We used the standard method of Maximum Likelihood (ML), as given for example in Cheng [6], to estimate parameter values.

Here we outline the approach (fitting the model) used to estimate the parameters from a sample of observed daily deaths, this being used to prepare Fig 1 in the main text. Let the sample of observed number of daily deaths be denoted by

$\mathbf{Z}=\{z_{t}t=1,2,\ldots T\}$ (7)

where $z_{t}$ is the number of deaths on day *t* and $T$ is the number of days observed. If the observations were made without error and if, with the right parameter values are correct for $\boldsymbol{\theta}$, then the death trajectory $\{Z\left( t, \boldsymbol{\theta} \right) t=1,2,\ldots,T\}$ would match the observed deaths **Z** in (7). So the model would then be successful in explaining deaths.

To include statistical uncertainty in the model we assume instead

$z_{t}=z\left( t,\boldsymbol{\theta} \right)+e\left( t \right) t=1,2,\ldots,T$ (8)

where $e(t)$ is random error. For simplicity the $e\left( t \right)$are assumed to be normally and independently distributed (NID) with standard deviation $\sigma,$ i.e.

$e(t$) ∼ NID(0,$\sigma$²), so that $z_{t}-z\left( t,\boldsymbol{\theta} \right)$∼ NID(0,$\sigma$²) (9)

The logarithm of the distribution of the sample is then

$L\left( \mathbf{Z} | \boldsymbol{\theta} \right)=- (T/2)ln(2\pi) -T\ln\sigma- [1/(2\sigma^{2})]\sum_{i=1}^{N} {{[z}_{t} - z(t,\boldsymbol{\theta})]}^{2}$ (10)

where $\mathbf{Z}$ is the random argument, and the parameters $\boldsymbol{\theta}$ are fixed. In ML estimation (MLE), this is turned on its head so that **Z** is simply the known sample of observations now regarded as fixed and we write L as $L\left( \mathbf{Z} | \boldsymbol{\theta} \right)\boldsymbol{=}L\boldsymbol{(}\boldsymbol{\theta}\boldsymbol{|}\mathbf{Z}\boldsymbol{)}$) calling it the (log)likelihood to indicate that it is now treated as a function of $\boldsymbol{\theta}$. The ML estimator $\hat{\boldsymbol{\theta}}$ is simply the value of $\boldsymbol{\theta}$ at which L($\boldsymbol{\theta}$|$\mathbf{Z}$) is maximized. i.e.

$\hat{\boldsymbol{\theta}}$ = $\mathrm{argmax}_{\boldsymbol{\theta}}${(L($\boldsymbol{\theta}$|$\mathbf{Z}$)} (11)

Nelder-Mead numerical search for the maximum was used. This goes through different $\boldsymbol{\theta}_{i}$ *i*=1, 2, 3,… comparing the different $L(\boldsymbol{\theta}_{i},|\mathbf{Z}$) to find $\hat{\boldsymbol{\theta}}$, the best $\boldsymbol{\theta}$.

To simplify description of the estimation process, only fitting to deaths data, **Z** as in (7) has been described, but the method extends straightforwardly to include other data samples. For example

$\mathbf{Y}=\left\{ y_{t} t=1,2,\ldots,T \right\}$ (12)

where $y_{t}$ is the number of active cases on day *t*. Fitting simultaneously to both **Y** and **Z** can be carried out by adding to the right-hand side of (10) a corresponding set of terms for **Y**. Numerical solution of the differential equations requires initial values for *S*, *E*, *I*, *R*. These are essentially scale invariant with $(S+E+I+R)$ constant and independent of *t.* So the numerical integration can conveniently be done using S(0, $\boldsymbol{\theta}$) = 1, E(0, $\boldsymbol{\theta}$) some small quantity subsequently adjustable as its initial value $e_{0}$ is a parameter; with *I* and *R* also initially zero. The size of the exposed population, parameter *s_0_*, is only needed to provide scaled values *S*, *E*, *I*, *R* at each step for comparison with the data **Y** and **Z**.

**Empirical model of epidemic growth and decline: skew-logistic**

The simple SEIR model above cannot accurately describe European COVID-19 epidemics when constrained by biologically plausible parameter values, notably the slow rates of decline in the asymmetric epidemics (although variations on the basic SEIR model could probably do so, e.g. relaxing the assumption of exponentially distributed delay times) [7, 8]. Without changing initial conditions, the number of people infected (and the number that die), and the timing of the epidemic, can be adjusted either by changing *R_0_* (Fig 1a) or by changing size of the exposed or susceptible population (Fig 1b). For example, to describe the epidemic in Germany (Fig 1), the SEIR model above assumes that only a fraction of a national population (*s_0_*) is exposed to or susceptible to infection prior to the introduction of COVID-19. The size of the epidemic is then determined by *R_0_* and by the rate of decline in *R_t_* as the susceptible population is depleted by the spread of infection. The alternative way to describe the European epidemics is to allow a change in initial conditions, in particular by reducing *R_0_* to *R_0c_* during the course of the epidemic, for example to represent the effect of non-pharmaceutical interventions) [3, 9, 10].

The SEIR model thus serves our purpose in Fig 1 of the main text. However, in order to avoid making strong assumptions *a priori* about the mechanism of epidemic control, we devised a flexible empirical model to describe the European epidemics. A number of functional forms (variations on the skew-logistic, the Shannon entropy index [11], and others) could probably describe these data but, for this study, we derived a new form of the skew-logistic:

$D\left( t \right)=a\frac{e^{g(t-\tau)}}{{[1+e^{\frac{1}{2}\left( g-f \right)\left( t-\tau\right)}]}^{2}}$ (13)

where *D(t)* is the number of deaths per unit time (although the model could apply to case incidence too). The model depends on just four parameters: *a*, a scaling parameter used to adjust the size of the epidemic; *g* and *f*, two slope parameters, one characterising the rate of rise, the other the rate of decline of the epidemic; and $\tau$, a location time parameter indicating when the epidemic is at its peak.

We assume that $g>0$ and $f<0$ so that the term $\frac{1}{2}\left( g-f \right)$ in the exponential expression in the denominator is always positive. Then $D\left( t \right)\cong e^{g(t-\tau)}$ if $t\to-\infty$, so that *g* gives the exponential rate of increase of the epidemic as *t* increases from $-\infty.$ However, $D\left( t \right)\cong e^{f(t-\tau)}$ if $t\to\infty$, so that $f<0$ gives the exponential rate of decrease of the epidemic as this subsides.

In addition, equation (13) is identical to

$D\left( t \right)=a\frac{e^{f(t-\tau)}}{\left( 1+e^{-\frac{1}{2}\left( g-f \right)(t-\tau)} \right)^{2}}$ (14)

So that *g* and *f* are mathematically identical, and either (13) or (14) can be used when fitting to data, with *g* and *f* playing either role depending on their sign.

There are two useful additional characteristics of *D*(*t*). First, it has the explicit maximum value

$D_{max}=ar^{(\frac{2r}{1+r})}{(1+r)}^{-2}$ (15)

where $r=-g/f$ Second, the maximum point is at

$t_{max}=\tau+\frac{2}{g-f}log(-\frac{g}{f})$. (16)

This shows that $\tau$ is close to where the epidemic maximum occurs, if *g* and $-f$ are comparable so that the second term in the right-hand side of (16) is near zero. In any case, *t_max_* indicates where the epidemic will peak; a convenient quantity for comparing the epidemics in different countries. In addition, we can measure the period of epidemic growth as the time from 1 death/day (estimated from the model fit rather than counted in the data) to *t_max_*.

Thus all four parameters have readily understandable, practical interpretations, and the model can easily be fitted to data (cases or deaths) using the method of maximum likelihood (ML), where we employ here Newton-Raphson numerical search. This latter needs initial parameter values to be provided. We do this by first examining the data to see if the epidemic appears to have peaked or not, and if so obtain an initial estimate of its position and size. This allows rates of increase and decrease to be roughly estimated to give starting values for *g* and *f*. An initial value for *a* can then be obtained using (15), whilst equation (16) shows that the observed maximum of the epidemic can be used as the initial value of $\tau$. Note that an extra parameter, *σ*, the standard deviation of the observational error, is included in the ML method, and this parameter also being estimated.

This skew-logistic model should not be mistaken for the established, similarly named, skew-logistic distribution and its generalizations, these latter being probability distributions that have different mathematical characteristics to (13). The new model is particularly suited to bell-shaped but asymmetric epidemic trends where there is a rapid increase to a peak before a decrease that is usually more gradual.

We fitted the model to data from 24 countries for which there were sufficient reported deaths (Figs 2 and 3, Fig S3), giving the ML estimates in Fig 2 and Table S3 (at the end of this document). Software to fit the model is available from the authors.

**Fig S3**. Estimates of the rates of epidemic growth and decline obtained from the skew-logistic model, illustrated for the United Kingdom. Data (points) are summed over 7 days, covering the weekly reporting cycle. The maximum growth rate of the epidemic is 0.34/day (the growth rate that determines *R_0_*) and the maximum rate of decline is 0.03/day. The fit is also shown in Fig 2 of the main text.

**Non-pharmaceutical interventions: policies on lockdown**

We characterized lockdown policies based on the Government Response Tracker created by the Blavatnik School of Government, Oxford [12]. The Containment and Health Index is based on eleven indicators including containment and closure policies (schools, workplaces, travel bans) and health system policies (information, testing, contact tracing), with a score for each country varying between 0 and 100%.

For each country, CHI increased with time (Fig S4) and, in general, later decreased (Fig 4b). Using this index, we calculated the cumulative number of deaths at lockdown (286 deaths in the UK) as the average number weighted by the magnitude of incremental step of CHI. The date of lockdown is the date corresponding to the average of the cumulative number of deaths (22-23 March in the UK).

**Fig S4**. Cumulative reported deaths (red) and the Containment and Health Index (blue) for the United Kingdom. The circle marks the number of deaths (286) and the corresponding date of lockdown (22-23 March).

**Fig. S5**. The relation between total deaths and deaths at lockdown based on CHI, as in Fig 4a of the main text. The power relationship (central line) is $D=162D_{L}^{0.73}$. Errors on the regression line are 95%CI (lateral lines).

This interpretation of the number of deaths at lockdown gives the univariate relation between total deaths (*D*) and deaths at lockdown (*D_L_*) shown in Fig S5, which is integral to the multiple regression in Fig 4a of the main text.

Alternative interpretations of the date of lockdown give similar results. One of these alternatives, among several permutations explored, is the number of deaths recorded when the Stringency Index (as for CHI, but excluding the health system policies) reached 70% (Fig S6) [12].

**Fig. S6**. An alternative interpretation of the timing of lockdown based on the Stringency Index applied to the 24 countries in Figs 2 and 3, yielding similar results to Fig S5. The power relationship (central line) is $D=203D_{L}^{0.81}$. Errors on the regression line are 95%CI (lateral lines).

**Table S3**. Parameter estimates for the skew-logistic model fitted to 24 countries.

The skew-logistic model is fitted to data on the number of deaths reported daily as these should have more independent and identically distributed random errors than 7-day moving averages (Table S3). Upper and lower confidence limits were obtained both by standard asymptotic theory and bootstrapping. Asymptotic theory tends to give more optimistic (narrower) confidence intervals (CI). Bootstrapping allows for asymmetry in the CI, which are wider.

The countries listed in this Table exclude Albania, Bulgaria, Iceland, Ireland, Serbia and Slovakia for which epidemics could not be described by the skew-logistic, either because there were too few data (e.g. Iceland), or the data did not describe a standard epidemic curve (Bulgaria).

**References**

1 Dye, C. 2020 The scale and dynamics of COVID-19 epidemics across Europe *Dryad Dataset* [**https://doi.org/10.5061/dryad.f1vhhmgv6**](https://doi.org/10.5061/dryad.f1vhhmgv6),

2 European Centre for Disease Prevention and Control. COVID-19. Solna: European Centre for Disease Prevention and Control 2020.

3 Flaxman, S., Mishra, S., Gandy, A., Unwin, H. J. T., Mellan, T. A., Coupland, H., Whittaker, C., Zhu, H., Berah, T., Eaton, J. W.*, et al.* 2020 Estimating the effects of non-pharmaceutical interventions on COVID-19 in Europe. *Nature*. **published online 9 June**, (10.1038/s41586-020-2405-7)

4 Ma, J. 2020 Estimating epidemic exponential growth rate and basic reproduction number. *Infectious Disease Modelling*. **5**, 129-141. (10.1016/j.idm.2019.12.009)

5 Dagpunar, J. S. 2020 Sensitivity of UK Covid-19 deaths to the timing of suppression measures and their relaxation. *Infectious Disease Modelling*. **5**, 525-535. (doi:10.1016/j.idm.2020.07.002)

6 Cheng, R. C. H. 2017 *Non-Standard Parametric Statistical Inference*. Oxford: Oxford University Press.

7 Wearing, H. J., Rohani, P., Keeling, M. J. 2005 Appropriate models for the management of infectious diseases. *PLOS Medicine*. **2**, e174. (10.1371/journal.pmed.0020174)

8 Royal Society. 2020 Reproduction number (R) and growth rate (r) of the COVID-19 epidemic in the UK: methods of estimation, data sources, causes of heterogeneity, and use as a guide in policy formulation. London: Royal Society.

9 Davies, N. G., Kucharski, A. J., Eggo, R. M., Gimma, A., Edmunds, W. J., Centre for the Mathematical Modelling of Infectious Diseases, C.-w. g. 2020 Effects of non-pharmaceutical interventions on COVID-19 cases, deaths, and demand for hospital services in the UK: a modelling study. *Lancet Public Health*. (10.1016/S2468-2667(20)30133-X)

10 Ferguson, N. M., Laydon, D., Nedjati-Gilani, G., Imai, N., Ainslie, K., Baguelin, M. 2020 Report 9: Impact of non-pharmaceutical interventions (NPIs) to reduce COVID-19 mortality and healthcare demand. (<https://doi.org/10.25561/77482>)

11 Dalziel, B. D., Kissler, S., Gog, J. R., Viboud, C., Bjornstad, O. N., Metcalf, C. J. E., Grenfell, B. T. 2018 Urbanization and humidity shape the intensity of influenza epidemics in U.S. cities. *Science*. **362**, 75-79. (10.1126/science.aat6030)

12 Hale, T., Webster, S., Petherick, A., Phillips, T., Kira, B. 2020 Oxford COVID-19 Government Response Tracker. *Blavatnik School of Government*.
